# Home as a Place of Refuge, Despair, and Self-Care for Men Living With Mental Health Challenges

**DOI:** 10.1177/10497323251320848

**Published:** 2025-03-19

**Authors:** Sarah K. McKenzie, Amy Cooper, Christina Ergler, Mary T. Kelly, John L. Oliffe

**Affiliations:** 1 Department of Psychological Medicine, 2495 University of Otago, Wellington, New Zealand; 2 School of Geography, 2495 University of Otago, Dunedin, New Zealand; 3 School of Nursing, 8166 University of British Columbia, Vancouver, BC, Canada; 4 Department of Nursing, 110600 University of Melbourne, Parkville, VIC, Australia

**Keywords:** men’s mental health, photovoice, masculinity, coping strategies, enabling places

## Abstract

Men’s poor mental health outcomes and heightened risk for suicide have been linked to their maladaptive responses to life stressors. In this photovoice study of 21 New Zealand–based men who experienced depression, anxiety, and suicidality, participants’ home emerged as an important place and resource for dealing with their mental health challenges. Utilizing the therapeutic landscapes “enabling places” framework combined with masculinities theory, we explored the affective, material, and social resources of home for determining how men’s mental health challenges play out behind closed doors. Reflexive thematic analysis was used to analyze individual photovoice interviews, revealing how home served along a continuum of enabling and disabling spaces. In this context, home could be a place of refuge, despair, and self-care for participants, and the specificities of those inhabiting forces are described thematically. In terms of *refuge*, the materiality of home created an affective sense of safety that afforded men spaces to privately conceal and deal with their mental health challenges. Home could also invoke *despair* in being a risky or disabling place where men felt trapped and isolated, heightening self-harm risks. These same spaces could also promote men’s *self-care* practices in the context of managing their anxiety, depression, and/or suicidality. The current study findings confirm the need for more research that is place and space based to inform mental health supports for men. Implications for men’s mental health promotion are discussed.

## Introduction

Coping strategies play an important role in how men manage mental health challenges, and are closely connected to their suicide risk levels (Bilsker et al., 2018). Men’s mental health literature has linked conformity to masculine norms with the uptake of maladaptive strategies during periods of distress (Garcia, 2016; Spendelow & Seidler, 2020). This includes alcohol and drug use, concealing and minimizing distress symptoms, risk-taking, and prioritizing self-sufficiency and independence rather than seeking professional help (Spendelow, 2015). Although such approaches may provide temporary relief from distress, for men with a tendency toward social isolation and withdrawal from relationships, these behaviors can increase vulnerabilities and suicide risk (Player et al., 2015).

While the predominant focus in the literature is on men’s maladaptive coping strategies, there is some research which has identified men’s proactive approaches to dealing with mental health challenges including accessing social support networks, physical activity, making lifestyle changes, and/or engaging in therapy to develop coping skills and resilience (Fogarty et al., 2015; Proudfoot et al., 2015). However, these positive strategies are frequently reported as emerging after a period of ineffective approaches and worsening, oftentimes severe symptoms (Whittle et al., 2015). Conspicuously absent from the men’s mental health literature is an explicit focus on the places and spaces where men manage their mental health challenges including the home—a key place in the everyday lives of many men who experience mental health challenges (Tucker, 2010). Addressing this knowledge gap, the current photovoice study explores how home is experienced and inhabited by men who experience anxiety, depression, and suicidality. We position our study at the intersection of health geography, masculinities, and men’s lived experience of mental health challenges, and in doing so an original and much needed contribution is made to advance the field of men’s mental health promotion.

### Connecting Place and Men’s Mental Health

The relationship between place, space, and mental health is well documented in the broader mental health literature (Curtis et al., 2010). Health geographers working with the key concepts of therapeutic landscapes have examined how the physical and social aspects of the environments in which people live may threaten or support mental health and well-being. From small scale ones (forest, gardens, health care settings) to large-scale physical environments (suburbs, coasts, urban spaces), researchers have emphasized the importance of exploring everyday places (Bell et al., 2018; Taheri et al., 2021). However, less is known about how the environments where men live, work, and spend their time can significantly impact mental health challenges and management. There is a small but growing body of work that considers the therapeutic potential of various environments to identify strategies for promoting men’s health and well-being across diverse settings (Heinz & Houghton, 2023; Herron et al., 2020; Ogborn et al., 2022). For example, the material aspects of everyday natural environments and outdoor spaces, such as backyards, farmland, golf courses, and waterbodies, can have therapeutic effects on mood, stress levels, and overall well-being by providing opportunities for relief, relaxation, recreational activities, and spaces for social connection (Ahmadu et al., 2021; Wilson et al., 2023).

The social aspects of spaces can also play a crucial role in supporting mental health by providing opportunities for social interaction, physical activity, and the formation of meaningful connections. For example, Men’s Sheds, community spaces for older men, provide non-threatening social spaces where men can perform and express their identities, [re]establish a routine and sense of purpose, and experience greater social inclusion (Anstiss et al., 2018; Kelly et al., 2021). Likewise, barbershops have been identified as spaces which may provide a safe and familiar setting where men can talk openly about personal issues and socially connect with other men (Ogborn et al., 2022).

Emergent qualitative research exploring men’s lived experience of mental illness suggests connections to place are more complex than providing arenas such as Men’s Sheds or tailoring everyday spaces like barbershops. Specifically, not all aspects of mental health promoting places and spaces are accessible to men, especially those who align to dominant masculine ideals prescribing self-reliance and stoicism. For example, masculine workplaces can shape men’s buy-in to norms that reward aggression, competition, and power, with negative effects heightening their risk for depression and suicide (Oliffe & Han, 2014; Seaton et al., 2017). Similarly, rural places have been connected to men’s poor mental health outcomes and struggles to cope with distress due to stigma and the policing of gender practices within remote masculine milieus (Creighton et al., 2017a; Herron et al., 2020). These studies illustrate the diverse relationships between place, space, and men’s mental health and illness, highlighting the importance of further considering geographies in men’s mental health.

### Home, Place, and Therapeutic Landscapes

There are few studies that explore the therapeutic role of home in men’s management of mental health challenges. Coen et al. (2013) drew attention to the importance of the home as a central place in men’s management of depression. In their study of Canadian rural-based men, Coen et al. (2013) highlighted how men and their female partners enacted coping strategies which deviated from dominant masculine rural norms to support men’s well-being. These coping strategies often occurred relationally and privately, for example, sharing domestic responsibilities, which Coen argues perpetuated an “invisible geography” of men’s depression.

Focusing on the role of home in how men cope with mental health challenges is important for several reasons. First, the home is a place where men spend a significant amount of time (i.e., it constitutes an everyday geography) linked to health and well-being (Mossabir et al., 2021), making it a key setting for intervening and supporting men to manage mental health challenges in their private lives. Second, home as an experiential place has been overlooked in geographical work on men’s mental health and place-based masculinities (Gorman-Murray, 2013). Third, home is a common location for suicide deaths among men, with almost 70% of all male suicides in New Zealand occurring at the deceased’s home, suggesting a role for place-based suicide prevention interventions (Suicide Mortality Review Committee, 2016). This study explores how home is operationalized and inhabited by men who experience anxiety, depression, and suicidality.

Several theoretical frameworks have emerged to explain how places become therapeutic or enabling for different groups. Expanding on the therapeutic landscapes concept, which suggests that certain settings and places possess properties that promote health (Gesler, 1992), Duff’s *enabling places* is a relational approach which seeks to understand how interactions between people and resources within a place can help or hinder health (i.e., how the “therapeutic” actually occurs) (Duff, 2011, 2012). A place may be regarded as an *enabling place* “insofar as it features networks and associations that generate the resources and agencies necessary for the maintenance of health” (Duff, 2011, p. 153). Duff (2012) breaks these resources down into material, social, and affective resources. Material resources are the most tangible therapeutic features, and include a diverse range of objects, assets, and benefits which contribute to the therapeutic utility of a place, by “facilitat[ing] the realisation of specific enabling or health promoting activities” (Duff, 2012, p. 154). Social resources are tied to social capital and the development of close and extended social networks or support structures and may include positive relationships and established rapport among individuals, along with a shared sense of trust and open communication. Affective resources underpin and stem from both material and social aspects and enable a person to alter their “affective state” and experience feelings of being enabled to utilize their “action potential” (Duff, 2011, p. 149). As such, affective resources are produced in place through encounters with other people (social) and the physical space (material) to evoke emotional experiences, feelings, and moods (Duff, 2011, p. 153). In the current study, *enabling places* provides a means to consider how the specificities of home—material resources, social relations, and affective dimensions—might account for how men manage their mental health challenges (Duff, 2011).

Studies using the enabling places concept have been conducted in diverse settings and populations including ageing in the community (Li et al., 2023), smoking in urban spaces (Tan, 2013), the built environment of cancer care centers (Martin & Roe, 2022), recreational sport and physical disabilities in youth (Bates et al., 2019), homelessness and substance use (Evans et al., 2015), drug use in marginalized neighborhoods (Ivsins et al., 2019), and those living with mental illness in the community (Hope et al., 2023). Findings from these studies suggest that health and well-being is enabled (or not) in diverse ways and settings (Duff, 2012). To our knowledge, the enabling places framework has not been applied to men’s mental health in the context of home. Thus, our analysis is sensitive to how men living with anxiety, depression, and suicidality experience and navigate the availability of these different resources in the home.

## Methods

Guided by a social constructivist approach (Creswell, 2014), we sought to understand men’s experiences of living with mental health challenges at home. Grounded in the understanding that realities are multiple and socially constructed, and knowledge is co-created, social constructivism guided the study design, incorporating in-depth interviews to capture participants’ narratives (Creswell & Poth, 2018; Fleuret, 2018). In line with constructivism, reflexive thematic analysis (Braun & Clarke, 2022) and the enabling places framework (Duff, 2010, 2011) were used to examine men’s experiences of home as resource for coping with mental health challenges.

Further, we used photovoice, a participatory action research method where men took and narrated photographs to share their experiences and perspectives (Wang & Burris, 1997). The methods and study design were chosen because we wanted to understand mental health challenges through men’s firsthand accounts, privileging their stories and disrupting the power dynamics that can emerge in research with men living in marginalizing conditions (Wang & Burris, 1994). The use of participant-produced photographs was intentional as it can provide a means to communicate hard-to-conceptualize experiences such as suffering, stigma, and recovery in the context of mental illness (Han & Oliffe, 2016). Photo-based methods are also particularly effective for exploring emotionally sensitive topics with men who might feel uneasy disclosing their mental health challenges or have difficulty articulating their experiences with illness verbally (McKenzie et al., 2024).

The analysis presented here is part of a larger photovoice project that explored men’s gendered experiences of anxiety, depression, and suicidality, and what helped or hindered their recovery (McKenzie et al., 2023). Our focus on home emerged from the predominance of men’s talk and photographs representing their mental health experiences in the context of home. The analysis sought to answer the inductively derived research question: “What are men’s experiences of mental health challenges at home?”

### Data Collection

The study was approved for ethics by the University of Otago Human Ethics Committee (Health). A three-stage process of consent ensured informed consent was given for research participation, taking photographs of others, and release of creative materials and researchers use of participant-produced photographs (Creighton et al., 2017b). Participants were able to choose whether their photographs were used only in the research interviews, and/or academic outputs and/or an online photo-exhibition. Participants were given opportunities to withdraw from the study at any time and for their photographs to not be used in dissemination. Participants were advised of the risks involved in sharing their photographs and the use of pseudonyms through the information sheet and during meetings with researchers. Photo release forms indicating informed consent and approval to have photographs, including faces, used were completed by all participants in the study.

The study was promoted online through social media sites including Facebook (New Zealand–based men’s groups), Twitter (University social media channels), and sent out in a weekly bulletin from the Mental Health Foundation of New Zealand (a national charity). Potential participants were invited to contact one of two interviewers by email or telephone to confirm their eligibility. Inclusion criteria were (a) 18 years of age or older, (b) living in New Zealand, (c) English-speaking, and (d) current or previous experience of mental health challenges (comprising anxiety and/or depression and/or suicidality comprising suicidal thoughts, plans, and attempts). A total of 21 men ranging in age from 23 to 62 years old (mean age = 37) and self-identifying as New Zealand/European (*n* = 7), Māori (the Indigenous peoples of New Zealand) (*n* = 7), Australian (*n* = 2), British (*n* = 2), and European (*n* = 1) participated. In terms of relationship status, participants identified as single (*n* = 5), partnered (*n* = 7), married (*n* = 6), and divorced/separated (*n* = 1). The men identified as heterosexual (*n* = 12), gay (*n* = 8), and bisexual (*n* = 1). The participants reported diverse living arrangements, including living alone (*n* = 3), couple living (marriage, cohabitation, or long-term partnership) (*n* = 5), family household where parents and children live together (*n* = 7), and shared housing/flatting (*n* = 6). Most participants (*n* = 17) reported a history of suicidal ideation with almost half (*n* = 9) indicating they had at least one suicide attempt. Men who self-disclosed current suicidality were excluded from the study and referred to mental health services to minimize potential harm. Most men (*n* = 19) had previously or were currently receiving professional mental health care.

The research interviews were conducted by the first author, a white female with a background in mental health and suicide research, and a male interviewer with extensive experience in interviewing marginalized groups. The male interviewer also identified as Māori (indigenous peoples of New Zealand) which was important in terms of ensuring our recruitment and data collection was culturally sensitive and that we fostered an environment where participants felt safe to openly share their experiences.

Eligible individuals met with one of the two interviewers over Zoom/phone to discuss the study objectives, explain the photovoice assignment, and detail the process for being interviewed about their photographs. Participants provided informed written or verbal consent and completed a short demographic questionnaire. We asked participants to take photographs on their cell phones illustrating their experiences of living with mental health challenges and to write a caption for each photograph which explained the meaning of the image. An individual photovoice interview was scheduled to occur within 2 weeks of participants enrolling in the study. At the photovoice interview, participants were invited to tell their stories using their photographs as a guide; the interviewer included loop and prompt questions to encourage participants to elaborate on the motivations and meanings behind each of their photographs (e.g., “what aspects of your mental health challenges are represented in this photo” and “what does this photograph mean to you?”). Interviews lasted 40–150 minutes, and participants submitted between 3 and 16 photographs each (*M* = 11). Interviews were conducted between 2021 and 2022 (over Zoom due to COVID-19 restrictions) with two interviews conducted in person. The interviews were digitally recorded, transcribed verbatim, and checked for accuracy. The 240 photographs were inserted to the transcribed interviews at the point in which participants discussed each of those specific images. Identifying information including names and places was removed and participant pseudonyms assigned by the researchers.

### Data Analysis

Data were analyzed using reflexive thematic analysis, an interpretive approach to qualitative data analysis that facilitates the identification and analysis of patterns or themes within a dataset (Braun & Clarke, 2022). We used a combination of both deductive and inductive coding (Braun & Clarke, 2019, 2022; Byrne, 2021) in which the three components of the enabling places framework—material, social, and affective resources—were brought *to* the dataset as well as themes inductively derived *from* the dataset. Data analysis was led by the female first author who conducted the interviews and worked in an iterative reflexive process throughout the analytic phases with the co-authors who have diverse backgrounds in public health, nursing, men’s health, and health geography.

In the first phase, the interview transcripts and corresponding photographs were read and re-read by two researchers to familiarize ourselves with the data. In the second phase, the transcripts were imported into NVivo qualitative data analysis software, where we initially focused on deductive coding aligned with the enabling places framework. We saw each participant’s experience of home as a process in which the three enabling components were encountered—material, social, and affective resources. We then worked inductively with the data, generating descriptive codes based on repeated readings of the transcripts, and our research question. These initial codes with corresponding data segments and photographs were subsequently exported into Microsoft Word documents to facilitate remote collaborative analyses. In the third phase, these documents were read independently by two researchers, and notes were made to document initial interpretations of the inductively and deductively derived codes. Each subset of codes underwent cross-review within the team to ensure consistency and comprehensiveness. This dual coding approach allowed for a robust analysis, balancing theory expectations with participant-driven insights. In the fourth phase, thematic mapping was used to organize and sort these initial codes into theme piles resulting in the development of three tentative themes: “safe but trapped,” “self-care and self-destruction,” and “social connections and expectations.” Finally, multiple rounds of theme development among the co-authors occurred to refine codes, merge overlapping ideas, and ensure alignment between deductive and inductive findings resulting in three themes—home as a place of (1) *refuge* details how the materiality of home created an affective sense of safety and concealment for men ameliorating their mental health challenges, (2) *despair* describes how home could lead men to feel entrapped with a heightened risk for self-harm, and (3) *self-care* where home could be a resource for men to alleviate their anxiety, depression, and suicidality. Though described separately, participants did not exclusively align to one pattern; rather, two or three themes could be present in each participant’s narrative. To develop the findings, we also incorporated a social constructionist masculinities lens to conceptually advance the ways in which men’s coping strategies shaped and were shaped by gender (Connell, 2005). As presented below, illustrative quotes were selected along with participant-produced photographs to convey the three themes relating to home and men’s mental health challenges. Pseudonyms were assigned to participants, and age along with other relevant details is provided with quotes when necessary to help orient readers to the speaker.

## Results

### Home as a Place of Refuge

Many participants described home as the only place they felt safe. They provided many photographs depicting the material elements of home, bedrooms, beds, drawn curtains, and closed gates, which they associated with safety. Objects and colors in the home could influence the ways in which places were experienced to produce and maintain feelings of safety. Home for many participants was perceived as protective because it was a place where they were not exposed to the gaze and judgment of others. This “safe” affective atmosphere was enabled by retreating within the physical confines of home, often bedrooms and closed curtains, as well as the social features of home, which offered privacy without public scrutiny or expectations to present a specific appearance or display particular behaviors.

Sean, a 37-year-old man with social anxiety who was the primary caregiver for his two children, shared Photograph 1, captioned *A Gated House.* Featuring a closed wrought iron gate at the bottom of his driveway, accompanied by a security camera, and he positioned these material objects as enabling him to feel less exposed.

I feel a lot safer now. Not safer from a threat, but safer from a social interaction, now that we’ve got the gate …, so people now have to stop at the gate and ring the bell.

**Photograph 1.**
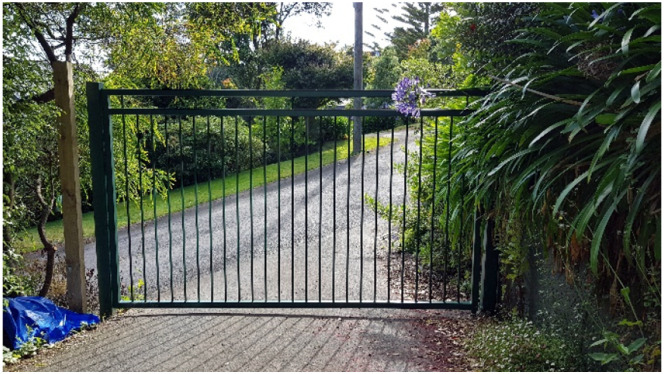
A gated house.

Sean used material elements at home as affective resources to create a boundary which defined and marked his home as separate from the outside world; the gate symbolized the restricted entry to his refuge. Home as a safe place was tied to his social anxieties about being in or infiltrated by the world. The gate ensured Sean’s home life was not disrupted by “surprise visitors” and afforded some control and concealment of his social anxiety. By having warning of unexpected visitors, Sean felt better able to limit his social interactions taking solace in his home being sheltered by a definitive boundary. He described feeling more “relaxed and comfortable,” experiencing fewer headaches, muscle aches, and fatigue and no longer feeling the need to take prescription medication. The private space of home also enabled Sean to be a stay-at-home-father—a role he valued, stating, “that’s my job.” Sean also viewed this domesticity as aiding his social anxiety in that he “didn’t have to go out.”

For many participants, the feeling of safety within the home was confined to their bedroom. Jason, a 39-year-old single man diagnosed with depression, shared Photograph 2 titled *Feeling Safe*, explaining how he spent long periods resting in bed to recover from his low mood and hide away his anxious mind.

This was just a depiction of quite a large season of my life which I just spend, you know essentially in bed, where I didn’t want to get out of bed, it felt safe being in bed, it felt safe being under a duvet, it felt you know, the room—I kept the curtains closed—you know but that was kind of how my mind was as well.

**Photograph 2.**
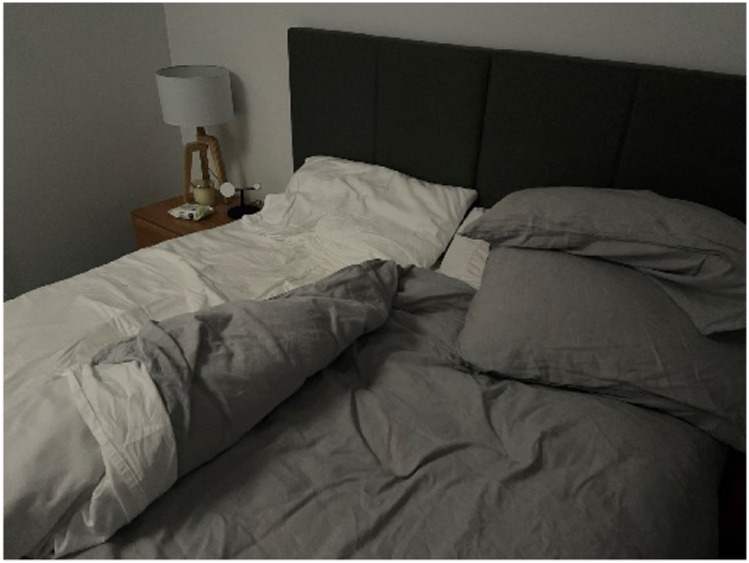
Feeling safe.

Jason created a sanctuary, a space of physical and emotional refuge, where he felt safe to process (or not) difficult emotions and back thoughts without judgment. Sharing a home with flatmates, the material elements of his bedroom—curtains, bed, door, and duvet—enabled Jason to create a small, protective space to also conceal from public view his depressive symptoms. While his closed curtains symbolized a separateness from the rest of the house (and its occupants) as well as the outside world, his duvet held him in a safe embrace. Evident in Jason’s narrative were masculine ideals around self-reliance and the avoidance of being seen as disabled and weak.

Home for most participants was built as a shelter—a place where their concealments held in abeyance the potential for others to judge them. This understanding of home is founded on the distinction between public and private, whereby the inside and outside world has previously been discussed by Mallett (2004). According to this binary, the inside or private sphere of home represents a comfortable, secure, and safe place, a refuge for when men were at their most vulnerable. In contrast, the outside was perceived as threatening, a space where control was lost, and relationality and interactions were outside participants’ control. This “safe” affective atmosphere was also generated through the materialities of home, which obscured public view and bypassed social expectations.

Brian, a 43-year-old sole parent to three young children who struggled with depression and social anxiety for many years, articulated in Photograph 3, titled *Hidden behind the mask*, his disguise to pass as being okay in public whenever he had to go out:

Most of my life, home is my safe place. I’m not agoraphobic or anything, being outside doesn’t bother me, it’s what’s in the outside that bothers me. It’s a bit like when people say I’m not scared of the dark, I’m scared of what could [be] in the dark. That’s kind of my relationship with outside.

**Photograph 3.**
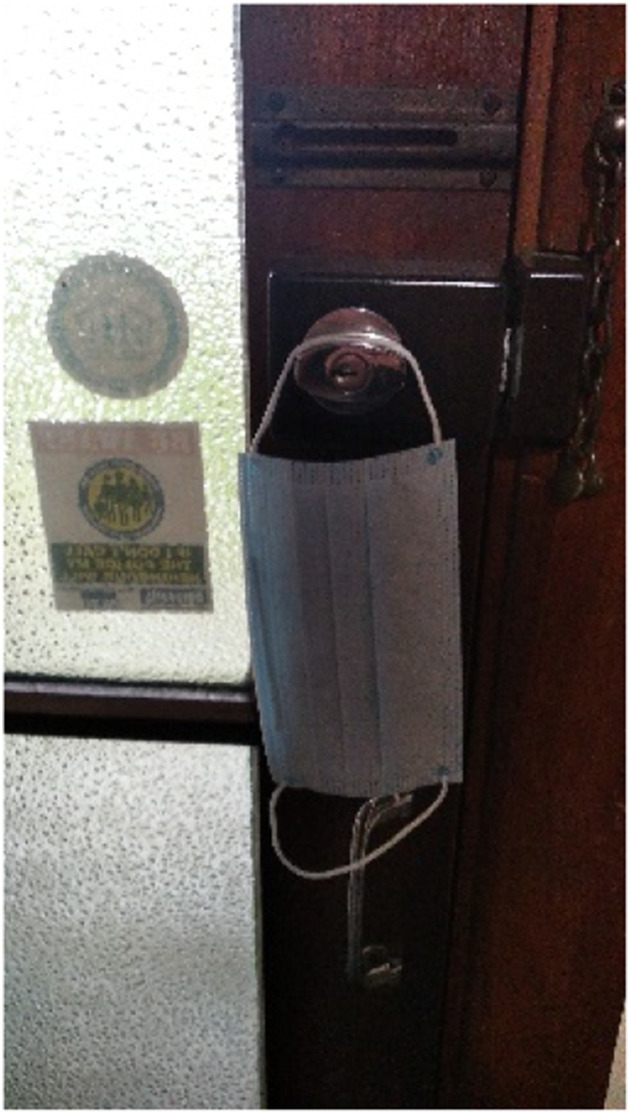
Hidden behind the mask.

Brian emphasized the uncertainty of being in public, suggesting he had a metaphoric mask to “put on a smiley face” and fake being an “outgoing and confident” man. Contrasting the safety and privacy of his own home, Brian took refuge in a place where he could be “vulnerable” and allow his true feelings to emerge without judgment.

Similarly, Pete, a 55-year-old man with chronic depression and anxiety, talked about feeling compelled to symbolically wear a mask at work: “I put on the mask at work but then afterwards, that would fall apart in some shape or form.” Pete explained that his mask was to conceal his “internalized stigma and the shame accompanying his depression and inability to pick himself up.” In contrast, home was de-stigmatizing in that he was able to take refuge from those pressures. Pete explained that he regularly wrote in his journal and talked to his family about “how I’m really feeling and thinking.”

In summary, home afforded affective and material resources using everyday objects and familiar settings to create a refuge to conceal men’s depression, anxiety, and/or suicidality. These hide away places afforded men spaces to sit with but not be seen and judged as embodying debilitating mental health challenges. The fear of public spaces underscored the refuge of home for many men.

### Home as a Place of Despair

For many men, home became a place of despair and confinement invoking isolation and a sense of not being able to escape. Participants’ emotional states were vividly expressed through their use of dark and bleak photographs. As a place of despair, men’s level of mental distress was entwined with the affective, material, and social resources available to them.

Staying at home featured prominently in the men’s photographs as did the awareness of some tensions wherein home was a place of confinement but was often depicted as relatively easier or safer than other environments. Will, a 23-year-old single man with depression, narrated Photograph 4 titled *Trapped*, sharing his experience of home as a disabling place entrapping him and his depression.

That’s just me sitting in my room during the day on my computer probably playing video games, probably vaping and then it being a perfectly good day outside and I feel like that is often where my depression traps me. I know that I should go outside but not having a reason to and not being able to find the motivation to leave the house. Feeling psychologically trapped in my room.

**Photograph 4.**
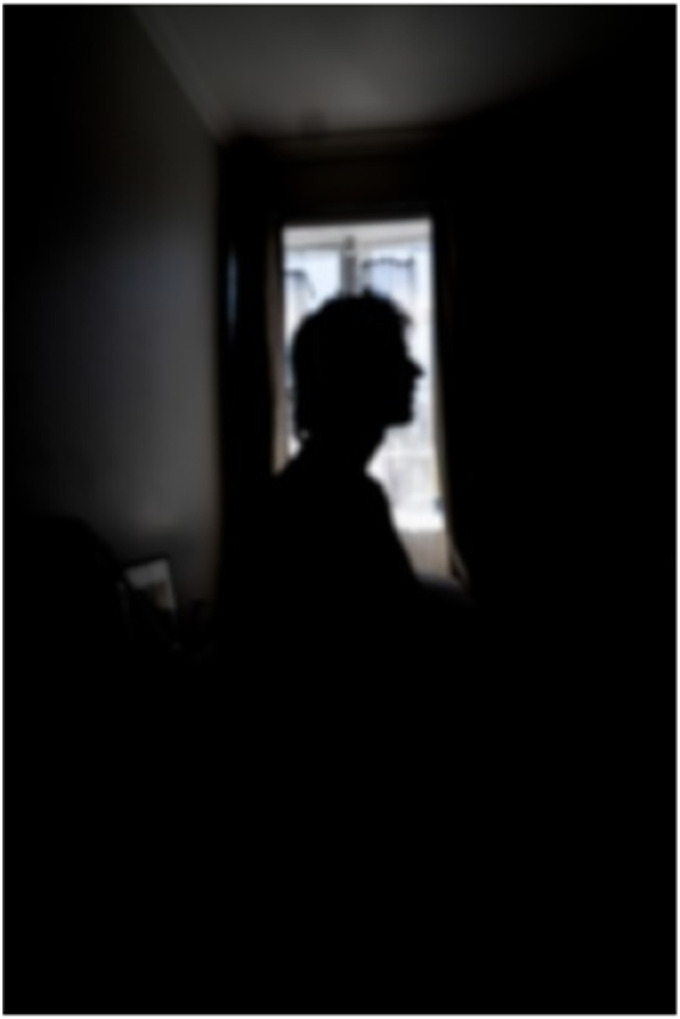
Trapped.

The self-silhouette depicted Will’s stillness centered with the window affording the only light in the dark space. Solitary and somber, Will explained he felt unable to leave his room or the home, expressing self-condemnation for choosing to stay inside. He acknowledged that staying indoors “jeopardized any chance of being successful,” conceding his debility state as marginalizing and without purpose. Will’s comments highlight how enmeshed men’s mental health can be with the social pressures to achieve normative masculine ideals associated with being a man, such as career and financial success. The material resources at home (i.e., vaping and gaming) represented affective needs for doing something other than his rumination and low moods. However, those reliances did little to quell what he was feeling—including his resignate state for failing to embody an idealized masculinity.

Similarly, for Tom, a 27-year-old man, home could also be a disabling experience, with his depression often leaving him unable to physically move:

I would have loved to have gone for a run and you know really activated the things that make me happy, but I just couldn’t move, couldn’t be bothered, and because [name of partner] wasn’t around I was allowing myself to stay in that trap.

Tom’s aloneness manifested fatigue and lethargy leaving him feeling psychologically and physically trapped. Being “trapped” at home was also accompanied by guilt and shame with some blame implicitly assigned to his context—being alone. With the absence of his partner, Tom was unable to harness the social and affective resources which he regarded as essential for helping him to move out of his entrapped state and pursue strategies that would lift his low affect and anxious mood.

Home was also a site of risk and self-harm for men. Access to material resources such as razors, food, drugs, and alcohol changed the ways in which participants experienced home. Tom shared an account of how he coped with his mental health challenges after a relationship breakup by smoking synthetic weed in his bedroom, a normative masculine recreation used to blunt painful emotions as previously described by Oliffe et al. (2022). Soon he was smoking every night:

You would just like zombie over to a couch and pass out … when we got paid, we would get straight to the sex shop, it was very expensive, $80 a bag, and you’d smoke a bag in a night.

Synthetic drugs provided Tom an affective resource to blunt his distress at a time when he lacked the resources to effectively manage what he was feeling. These maladaptive practices however contributed to Tom losing his apartment amid accruing thousands of dollars in debt. While men often attempted to alleviate and conceal their anxiety, depression, and/or suicidality by self-medicating at home, they also eventually realized how these strategies were disabling. Stuart, a 45-year-old man narrated Photograph 5 titled *Wine numbs everything*, lamenting his difficulty in effectively self-managing his mental health:

I’d say that, probably, the biggest hindrance to my anxiety would be not being in a routine, and not doing my exercise, not doing my meditation, not eating well, drinking too much, partying too much … all those things would contribute to being a hindrance to my wellness, and contribute to my anxiety.

**Photograph 5.**
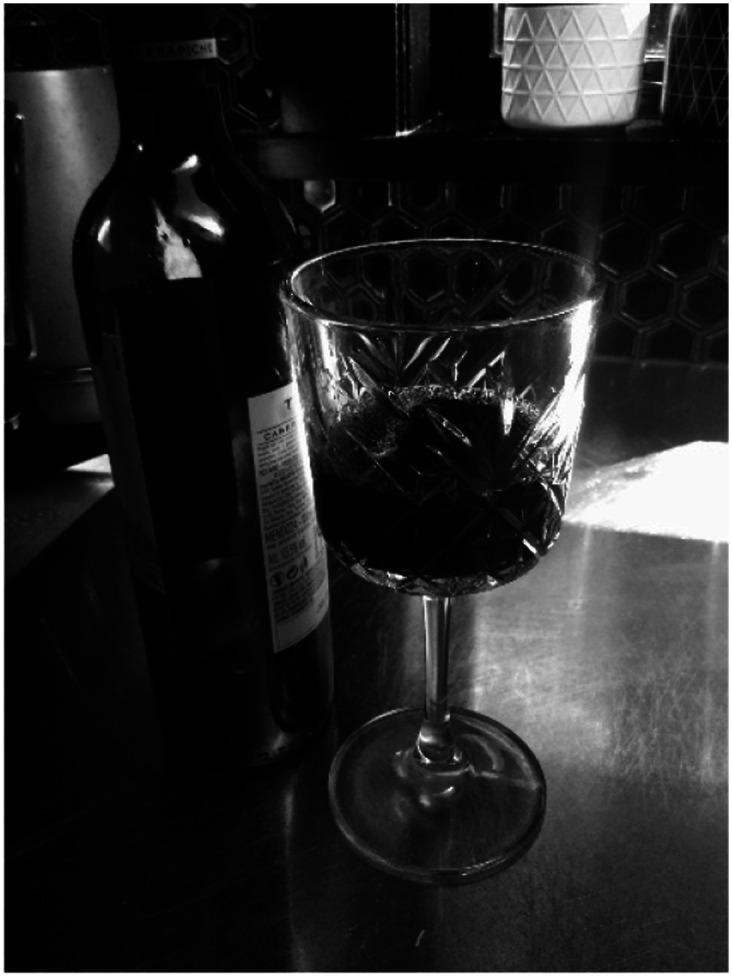
Wine numbs everything.

For Stuart, home was disabling place where he would get drunk every night. Aligned to masculine norms for men dousing what is uncomfortably felt, these at-home practices were usually solitary and carried significant isolation and risk for self-harm.

The home as a place of despair was closely tied to men’s isolation and self-harm risk. Sam, a 25-year-old man diagnosed with post-traumatic stress disorder (PTSD) and generalized anxiety, had made the decision to stop drinking alcohol because it prompted flashbacks to being sexually abused when he was a child. Often self-isolating at home, he recounted an event when he agreed to go out with friends as the designated sober driver. He said he was surprised at how much he enjoyed the night out “but then as soon as I went home, it was back.” Speaking about Photograph 6, *Darkness*, Sam explained this was his view from the backdoor steps of his home just prior to taking an overdose:

I was going through the motions in my head of, whether or not life was worth it. I took that picture at the time because it was, I don’t know, in one way I felt like those steps, were the way to do it. I could just throw myself off and fall and hope … I can still feel in a way, the pain I was carrying in that moment.

**Photograph 6.**
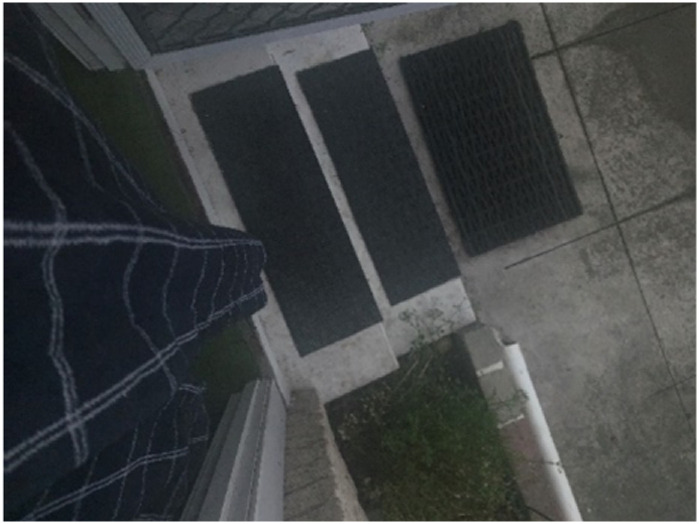
Darkness.

Sam’s quote and photograph speak to the affective atmosphere of home and disabling dimensions for ruminating past traumas. Evident in this and many narratives was how without the social and affective resources needed to arrest their mental health challenges, men could become suicidal. Overwhelmed by his pain, Sam said, “In my unwell state I made the decision that it [living] wasn’t worth it.” Like Sam, several men described a range of material resources in the home that afforded the means and time to plan suicide attempts. For these men, the absence of social resources—friends, family, or partners—at home from which they might have leveraged support, connection, and intervention heightened their risk for suicide.

The home as a place of despair fuelled men’s risk. A place where men felt trapped, isolated, and vulnerable to an array of self-harm ways and means was worrisome.

### Home as a Place of Self-Care

Many participants depicted home as a place of self-care where they could practice behaviors which quelled their anxiety, depression, and/or suicidality. For some men these included rituals or hobbies, for others they were positioned as affective self-care skills adapted to being at home. Most self-care practices were tactile in nature: gardening, renovating, woodworking, art, puzzles, cooking, or journaling, the parts and sum of which bolstered their mood. For example, Richard, a 62-year-old man, was diagnosed with PTSD and experienced persistent anxiety attacks after being made redundant at work and harassed and bullied by local neighbors. Working in his garden drew therapeutic value, and when he handled the plants and soil, Richard was able to better manage his mental health challenges. Sharing Photograph 7, titled *Natural growth, variability, and adaptation*, Richard explained:

If I just go in the garden, I know that I feel good. I can actually be in my house, and I can feel stressed by remembering things or I’m trying to do some work or just feeling annoyed by remembering things and starting to spiral. I know that I just need to go out in the garden.

**Photograph 7.**
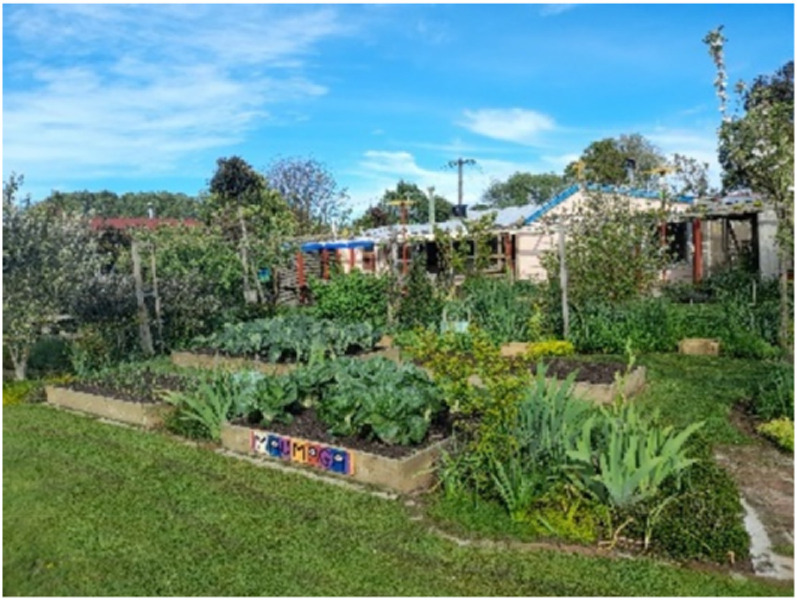
Natural growth, variability, and adaptation.

For Richard, his home extended to his garden, offering an intermediary and purpose for being both in and out of those spaces. Unfortunately, his neighbors objected to his gardening projects and lodged complaints with police, an example of the bullying he endured; however, Richard was undeterred as his garden provided him with an affective resource; it made him “happy.” He went on to explain how the growth in his garden was inspiring and healing for him, symbolic of his growing potential for survival and recovery. Richard embraced and valued the positive emotions that flowed from these self-care practices.

For fathers, caregiving and domestic responsibilities often restricted their social resources and the actions they were able to take to remedy anxiety and depressive symptoms. Consequently, men often described doing activities associated with their children as beneficial for their mental health. Here, social resources were not a direct interaction but more an affective resource to provide for their children. Brian, a 43-year-old acknowledged that his depressive symptoms were often difficult to manage as a solo father and shared Photograph 8 titled *Keeping busy and accomplishing.*

I mean just being a solo parent of three little people who have these enormous wants and needs that you meet as best you can, and not really having any time for me and not sleeping properly … I just enjoy building things for them [children] … I think for me it’s accomplishment in things but it’s also about fun through play … like shared joy maybe.

**Photograph 8.**
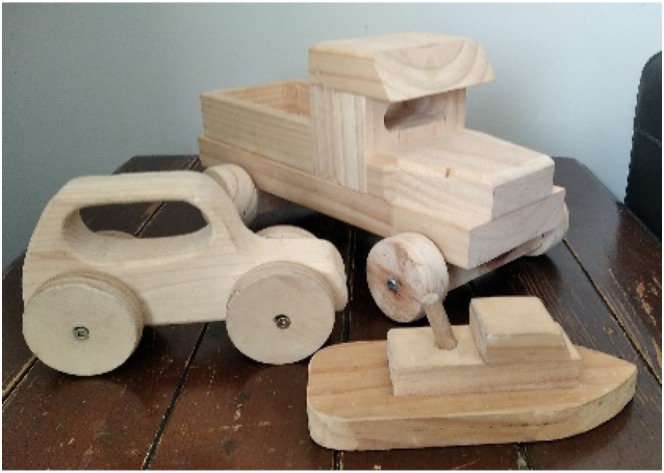
Keeping busy and accomplishing.

For Brian, these ameliorative activities were something he could do at home as self-care in that it would “keep my thoughts from wandering.” Doing something for his children also brought him a sense of providing, with the tactile nature of what he built a tangible product and pleasure of that labor. For these men, whose everyday life rarely afforded moments of stillness or relief from their pressures, therapeutic activities that resided both in and outside of traditional masculine pursuits (i.e., cooking, puzzles, reading, and arts) often provided ways of overcoming anxious thoughts and low moods. Becoming absorbed in such activities reduced ruminations and worries about the past or future, fostering presence and calm.

Social resources in the home were also therapeutic, providing affective benefits. Several men provided photographs of their dogs and talked about the healing power of pets. Sam, a 25-year-old man who had made three suicide attempts, relied on his dog Mya to comfort him during trauma-induced flashbacks. He referred to the dog as an “unconditional love machine,” and when he was crying or shaking with distress, “always without fail, she’d come, and she’d make sure I was okay. She’d start licking my face and that would snap me out of it.” Similarly, Ben, a 32-year-old man diagnosed with anxiety and depression, shared Photograph 9 titled *Best mate:*

Whenever you’d a bad day, you can always go [home] and see Izzy and that would always make me feel better, so I thought that was a good photo that captured how important she is to my mental wellbeing.

**Photograph 9.**
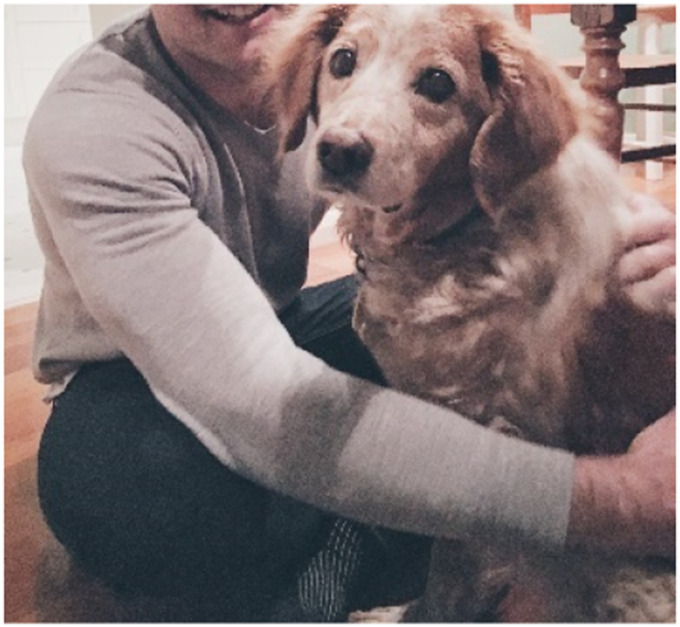
Best mate.

For Ben, Izzy was essential to his mental health, enabling him to “stay present” during periods of increasing distress related to paid work and expectations outside of his home. We observed that pets were posited as an essential non-human element in the home, providing many men with a therapeutic tactile experience to “ground” themselves as well as a sense of social connection that was free of judgment or reminders about demands invoked by the outside world.

The positive affective influence of partners and children also brought joy and mitigated negative emotional states for many men. The presence of loved ones in the privacy of home provided a place where men could experience and express their emotions without transgressing masculine norms of strength and stoicism as McKenzie et al. (2018) have previously detailed. Pete, 55 years old, described how his wife enabled him to cope because she was “just willing to be there when I’m feeling my most distressed.” Richard, 62 years old, explained how he felt grateful for having his son live with him and stated, “That is really what keeps me going. If I didn’t have that, life would be a lot darker I think because sort of I’ve got things to do I have to do.” Similarly, Sean, a 37-year-old man with social anxiety, said, “If I didn’t have my family, I would probably start to get more reclusive.” Many narratives and photographs featured significant others in the men’s lives and demonstrated how partners and children were an important enabling social and affective resource for sustaining men’s mental health with deep therapeutic effects.

In summary, men’s self-care practices at home were shaped by a variety of resources ranging from working with their hands and materials through to pets, partners, and children. These resources enabled participants to shift their introspection to others to derive a sense of purpose—one which worked to build positive emotions and affective relations in their home lives.

## Discussion

The findings drawn from the current study illustrate the importance of the home environment for men grappling with depression, anxiety, and/or suicidality to contribute place-based understandings about the role of the home in men’s mental health challenges. To be clear, these insights reveal what can go on behind closed doors to add to a vibrant emergent scholarship in men’s mental health and masculinities (Herron et al., 2020; McKenzie et al., 2023; Sharp et al., 2022). Evident in the participants’ interviews was the positioning of home, not merely as a physical location, but as a dynamic setting whose therapeutic nature changed depending on participants’ well-being. Whether home was a therapeutic place which could lift men up, a disabling place which could mire men down, or somewhere in between—a refuge—was dependent on men’s capacity to interact and navigate enabling resources at home. In other words, men’s ability to cope with mental health challenges was reliant on both access to an array of resources in the home and their current mental health state. Conceptualized in this way allows for an understanding of home as place of flux for men living with mental health challenges; a place which is experienced as both giving and taking from men’s mental health. These findings resonate with the broader health geography literature which highlights how particular settings can at times be places of safety and well-being and at other times places of risk and harm (Espeso, 2022; Ivsins et al., 2019; Lowe & DeVerteuil, 2022; Shortt et al., 2017). Our study offers several new insights into the relevance of home in the men’s mental health context.

First is the contradictory nature of home as both beneficial and detrimental for men living with depression and anxiety. In the context of mental health, the home is often perceived as a sanctuary, a place of comfort and security where individuals can seek solace and recharge from the demands of daily life (Blunt & Dowling, 2022). However, places are dynamic and can have diverse effects, on a continuum from enabling to harmful, due to the unique convergence of resources (Duff, 2012). For men in our study, home was a complex and sometimes contradictory place of refuge and despair, safety and risk, and self-care and self-harm. Whether explicitly or tangentially, participants connected the significance of their homes as a place of safety and security to the specific feelings and emotional states being at home evoked. Home became an “affective sanctuary” (Bell et al., 2018; Doughty, 2023), a place where men could retreat from public view concealing their mental illness, and seek out solitary time to reflect and nurture their emotions and mental health without the fear of stigma, judgment, or gendered social expectations. At the same time, the enabling resources of home could become disrupted leaving men feeling entrapped by their mental health challenges and the confinement of home. Our results extend findings on the ambiguous nature of home for precariously housed mental health service users (Lowe & DeVerteuil, 2022) and the therapeutic nature of home in panic disorder recovery (Espeso, 2022) by adding a gendered focus on men living with depression and anxiety.

Second, home was inhabited by an array of spaces, the most prominent being the embodied space of the bedroom. Ranging from the material resources, such as closed curtains and beds, to affective resources such as a sense of emotional refuge, these “enabling resources” were significant in supporting men’s coping with mental health challenges. However, these resources also supported men’s masculine self-reliance and concealment, contributing to men’s experiences of social isolation and increased risk. For participants in this study, the bedroom often became what geographers have referred to as a “third place” (Bell et al., 2018; Espeso, 2022) away from the social and gendered pressures of home, for example, flatmates, children, domestic responsibilities (the first place), or paid work (the second place). This experience was reported by men across all living arrangements including living alone, couple living, family household, and shared housing. This finding is significant given men’s withdrawal from family and friends has been shown to increase social isolation and risk of suicidality (Oliffe et al., 2018; Player et al., 2015; Ramirez & Badger, 2014). It is therefore important that families and those who live with men are supported to better understand and question men’s withdrawal to a “third place” within the home when struggling with mental health issues, especially those who live alone. Given isolation and being single are known risk factors for male suicide, this finding is critical for shaping effective male suicide prevention work (Bennett et al., 2023). There are also implications for mental health practitioners who should ask men about their home life and how they spend their time at home as key information in evaluating and treating men.

Third, home enabled men’s maladaptive coping strategies including drug and alcohol use, self-harm, and suicide attempts. Studies have consistently shown that men self-medicate with drugs and alcohol to mask their distress because of the shame associated with discussing mental illness and the risk of transgressing masculine ideals of strength and independence (Oliffe et al., 2018; Player et al., 2015; Ramirez & Badger, 2014). These practices in turn can lead to estrangement from family, friends, and professional mental health care, increasing men’s social isolation (Oliffe et al., 2018). We observed that home emerged as a site of suicidal behaviors for men when material resources (i.e., alcohol, drugs, prescription medication, and knives) converged with affective resources (i.e., feelings of confinement, despair, and isolation) and a lack of social resources (i.e., partners, family, and friends). Our findings offer a fresh perspective on how home can be a risk environment for men—a space in which a variety of factors interact to increase the chances of harm (Duff, 2010) and resonate with the broader geography literature which demonstrates *how* places that are not inherently risky can become so (Ivsins et al., 2019; Shortt et al., 2017). The current study suggests there is a need for placed-based mental health interventions that target men in the home and provide flexible and pragmatic solutions to respond to their real-time experiences and needs, for example, behavioral strategies men can use to improve their mood and manage distress; strategies to engage immediate social networks to intervene and help men reduce alcohol and substance use when they are feeling low; or e-mental health interventions which men can access anytime from anywhere. There are also implications for health practitioners who should ask men if they are drinking or using substances alone and work with men to advise against solo drinking as a harm reduction strategy.

Finally, our findings highlight the importance of men’s self-care practices and behaviors which played a crucial role in men’s efforts to gain or sustain stable (or at least tolerable) mental health. As an enabling place, home afforded diverse material, affective and social resources from everyday objects such as pets and houseplants to activities such as journaling, art making, cooking, and renovating to enable men’s coping. These findings support and extend research on men’s use of positive coping strategies (Fogarty et al., 2015; Proudfoot et al., 2015; Spendelow, 2015) by offering a place-based analysis of men’s self-care practices. For example, men valued the presence of pets in the home which facilitated non-demanding social interactions, enabling men to construct a caring masculinity and nurture their emotional well-being. As Elliott (2016) argues, caring masculinities can enrich men’s lives emotionally, psychologically, and physically. Men also found comfort in caring for house and garden plants suggesting that nature’s positive effects can also manifest on a much smaller scale. An interesting juxtaposition existed between men’s photographs of lying in bed versus doing and making things. These self-care practices often reflected interventions in the field of men’s health, especially the Men’s Shed movement that is based on the notion that men prefer “doing” activities, and the act of building things can offer therapeutic value to men (Oliffe et al., 2020). However, as a therapeutic landscape for self-care, home becomes problematic for men who are underhoused or lack the physical space or gardens to engage in the self-care activities described here; similarly, pet therapy demands the space and resources to care for an animal. An increased focus on men’s self-care practices and caring masculinities is of particular importance in the field of men’s mental health due to documented issues relating to men’s uptake of psychological treatments (Seidler et al., 2018). The field of men’s mental health promotion would benefit from considering the role of non-human elements, that is, the affective and material dimensions of enabling places (Duff, 2011) in men’s gendered coping with mental health challenges.

Our study also adds to the significant body of literature on the meaning of home—how people relate to and subjectively experience home (Blunt & Dowling, 2022; Easthope, 2004; Fox O’Mahony, 2012; Mallett, 2004). For example, home can provide a sense of self, security, safety and well-being, the presence of meaningful relationships, and space for activities beneficial for well-being (Fox O’Mahony, 2012; Mallett, 2004). However, the meaning of home is often centered on people’s positive bonds with home and the restorative potential of home environments for sustaining well-being (Aksel & Imamoğlu, 2023; Boulton et al., 2022; Kylen et al., 2019; Meagher et al., 2024) with less attention to the negative aspects of home. Our findings provide an important contribution to understanding that home is not always a positive environment for men and can become a place of negative impacts in which distress or illness amplifies. For men in our study, their relationship to home was complex and at times contradictory, both beneficial and detrimental to mental health. Our study also adds to the scholarship on “the home” in relation to gender (Gorman-Murray, 2012; Gorman-Murray & Hopkins, 2014), identifying intersections between masculinities and men’s coping with mental ill health within the private sphere of home.

In terms of limitations, the study’s reliance on a New Zealand context means the findings emerge from a specific local and temporal context that may not represent other men’s experiences, limiting their transferability to international/non-Western men. Limitations inherent to the methodology should also be considered when interpreting the current study findings. As all but two interviews were conducted using Zoom due to COVID-19 restrictions, the issue of selection bias should be considered. It is possible that the sampling method excluded men who were more socially isolated, living in precarious housing and/or socioeconomically deprived. We note that one interview took place in person due to a lack of access to a computer. While photovoice facilitated the discussion of in-depth personal experiences, a small number of interviews were shortened due to connectivity issues. This resulted in a wide variation in the length of interviews. Finally, limitations on the types of photos that can be taken in photovoice, such as not being able to take photos of people’s faces without their informed consent, may have inadvertently limited the men’s talk about the social aspects of their home environment.

Balancing these limitations, our findings add to an emergent body of photovoice work demonstrating the value of visual methods for engaging in discussions which provide insights into the diverse life worlds of men (Ferlatte et al., 2019; McKenzie et al., 2023; Oliffe et al., 2021). Taking photographs allowed participants to document aspects of the environment that enabled or hindered their coping with mental health challenges within the privacy of their own homes. As reported by Oliffe et al. (2023), men being in their home environments while being interviewed aided the flow, openness, and ultimately the richness of what was said and shared by participants.

A further consideration is that our study focused on the material, affective, and social resources available to men in the home from a mental health promotion frame. In relying on the enabling places framework, we are of course limited in what we are able to say about other aspects of men’s diverse relationship with home, for example, the relationship between home and men’s identity, self-expression, or sense of belonging (Fox O’Mahony, 2012; Tanner et al., 2012) or the cultural meanings of home for indigenous men (Boulton et al., 2022).

However, these limitations can guide future research to further describe men’s relationship with home and mental health. Future research would benefit from purposefully sampling specific subgroups of men with mental health challenges further exploring the themes of refuge, despair, and self-care, to test and compare these insights. Future examination into the influence of diverse living arrangements on men’s mental health would enable a deeper discussion on the significance of home for men experiencing mental health challenges. In our work, the isolation for men who lived alone, were solo parents or single, and living in shared rental arrangements starkly contrasted the social resources available to men who lived with partners or family. The relationships between home and mental health may also be disrupted, changed, or lost when partnerships or families are disrupted. Given the high rates of suicide among men, research exploring the role of affective and material resources in suicide prevention strategies targeting men who lack social resources within their homes would be worthwhile.

## Conclusion

An emerging body of empirical research has pointed to the importance of considering place-based interventions for men’s mental health, yet the home as a site of intervention for supporting men’s mental illness has remained under-researched. Our study revealed that photovoice is a powerful way for men to share insights into the resources within their homes which enabled (or disabled) their abilities to manage depression, anxiety, and suicidality. Participants experienced home as both enabling and disabling in the context of mental health challenges. As such, the current study findings serve as a call to action for families, friends, and mental health professionals to pay more attention to men’s lives behind closed doors, in order to address and reduce men’s anxiety, depression, and suicide.
